# Interaction of Cupidin/Homer2 with two actin cytoskeletal regulators, Cdc42 small GTPase and Drebrin, in dendritic spines

**DOI:** 10.1186/1471-2202-10-25

**Published:** 2009-03-24

**Authors:** Yoko Shiraishi-Yamaguchi, Yumi Sato, Rieko Sakai, Akihiro Mizutani, Thomas Knöpfel, Nozomu Mori, Katsuhiko Mikoshiba, Teiichi Furuichi

**Affiliations:** 1 Laboratory for Molecular Neurogenesis, RIKEN Brain Science Institute, Wako, Saitama 351-0198, Japan; 2 Department of Anatomy and Neurobiology, Nagasaki University School of Medicine, Nagasaki, Nagasaki 852-8523, Japan; 3 Laboratory for Neuronal Circuit Dynamics, RIKEN Brain Science Institute, Wako, Saitama 351-0198, Japan; 4 Laboratory for Developmental Neurobiology, RIKEN Brain Science Institute, Wako, Saitama 351-0198, Japan; 5 JST, CREST, Chiyoda-ku, Tokyo 102-0075, Japan

## Abstract

**Background:**

Homer is a postsynaptic scaffold protein that links various synaptic signaling proteins, including the type I metabotropic glutamate receptor subunits 1α and 5, the inositol 1,4,5-trisphosphate receptor, Shank and Cdc42 small GTPase. Overexpression of Homer induces changes in dendritic spine morphology in cultured hippocampal neurons. However, the molecular basis underpinning Homer-mediated spine morphogenesis remains unclear. In this study, we aimed to elucidate the structural and functional properties of the interaction between Cupidin/Homer2 and two actin-cytoskeletal regulators, Cdc42 small GTPase and Drebrin.

**Results:**

Cupidin/Homer2 interacted with activated Cdc42 small GTPase via the Cdc42-binding domain that resides around amino acid residues 191–283, within the C-terminal coiled-coil domain. We generated a Cupidin deletion mutant lacking amino acids 191–230 (CPDΔ191–230), which showed decrease Cdc42-binding ability but maintained self-multimerization ability. Cupidin suppressed Cdc42-induced filopodia-like protrusion formation in HeLa cells, whereas CPDΔ191–230 failed to do so. In cultured hippocampal neurons, Cupidin was targeted to dendritic spines, whereas CPDΔ191–230 was distributed in dendritic shafts as well as spines. Overexpression of CPDΔ191–230 decreased the number of synapses and reduced the amplitudes of miniature excitatory postsynaptic currents in hippocampal neurons. Cupidin interacted with a dendritic spine F-actin-binding protein, Drebrin, which possesses two Homer ligand motifs, via the N-terminal EVH-1 domain. CPDΔ191–230 overexpression decreased Drebrin clustering in the dendritic spines of hippocampal neurons.

**Conclusion:**

These results indicate that Cupidin/Homer2 interacts with the dendritic spine actin regulators Cdc42 and Drebrin via its C-terminal and N-terminal domains, respectively, and that it may be involved in spine morphology and synaptic properties.

## Background

Homer is a scaffold protein that is targeted to the postsynaptic density (PSD) of excitatory synapses. There are three distinct members, Homer1, Homer2 and Homer3, in this protein family [1-6] (for review see [7]). Postsynaptic Homer scaffolds interact with a variety of PPxxF (Pro-Pro-x-x-Phe) ligand motif-containing signaling molecules, including the type I metabotropic glutamate receptor subunits 1α and 5 (mGluR1α/5), the inositol 1,4,5-trisphosphate receptor (InsP_3_R) and Shank, via its N-terminal Ena/VASP homology 1 (EVH1) domain [1,2,4,5,8,9], and forms a tetramer by self-assembly via its C-terminal coiled-coil (CC) and Leu zipper (LZ) motifs [4,8,10]. In cerebellar Purkinje cells, the interaction of Homer3 with mGluR1α is regulated by activity-dependent phosphorylation at the linker region between the EVH1 domain and the coiled-coil domain [11]. In hippocampal neurons, Homer proteins co-cluster with the NMDA receptor complex during dendritic and synaptic differentiation [12], and regulate spine morphogenesis [13] as well as the functional organization of mGluR1α/5-InsP_3_R Ca^2+ ^signaling in dendritic spines [14].

Dendritic spine morphology is dynamically changed in response to synaptic activity, which is associated with synaptic functions including the long-term maintenance of synaptic strengthening [15,16]. Impaired spine morphology is known to contribute to mental retardations [15,16]. We previously showed that Cupidin, identical to Homer2, is co-sedimented with filamentous actin (F-actin) via the EVH1 domain, and also interacts with the GTP-bound, activated form of Cdc42 small GTPase via the C-terminal region [6]. Interestingly, over-expression of Cupidin/Homer2 suppressed Cdc42-induced formation of filopodia-like protrusions in HeLa cells [6]. Moreover, Cupidin/Homer2 was partly colocalized with Drebrin, a dendritic F-actin-binding protein, in the dendrites of cultured hippocampal neurons [12] and cerebellar granule cells [17]. It is known that both Cdc42 [18-20] and Drebrin [21,22] are involved in dendritic spine morphogenesis by regulating actin-cytoskeletal organization. A previous study showed that over-expression of Homer1b together with Shank induced enlargement of the spine heads of hippocampal neurons [13]. Together, the results of these studies suggest that Homer family proteins are involved in the regulation and/or plasticity of spine morphology by interacting with two dendritic F-actin regulators, Cdc42 and Drebrin. However, little is known about the molecular basis underpinning the involvement of Homer proteins in actin cytoskeleton-based regulation of spine morphology.

In this study we analyzed the structural and functional properties of the Cupidin/Homer2 scaffolding that interacts with two dendritic spine F-actin organization modulators, Cdc42 and Drebrin. We defined the Cdc42-binding domain in the C-terminal region of Cupidin/Homer2 and revealed the functional significance of Cdc42-binding domain in spine and synapse formation by cultured hippocampal neurons, as well as in Cdc42-induced filopodia-like protrusion formation in HeLa cells. We also proved Drebrin to be a Homer EVH1-binding target and showed the effect of Cdc42-binding domain on the Drebrin accumulation in spines. These results strongly implicate the postsynaptic Homer scaffolding in the morphogenesis of dendritic spines.

## Results

### Cupidin interacts with activated Cdc42 via the C-terminal coiled-coil region

**Figure 1:**
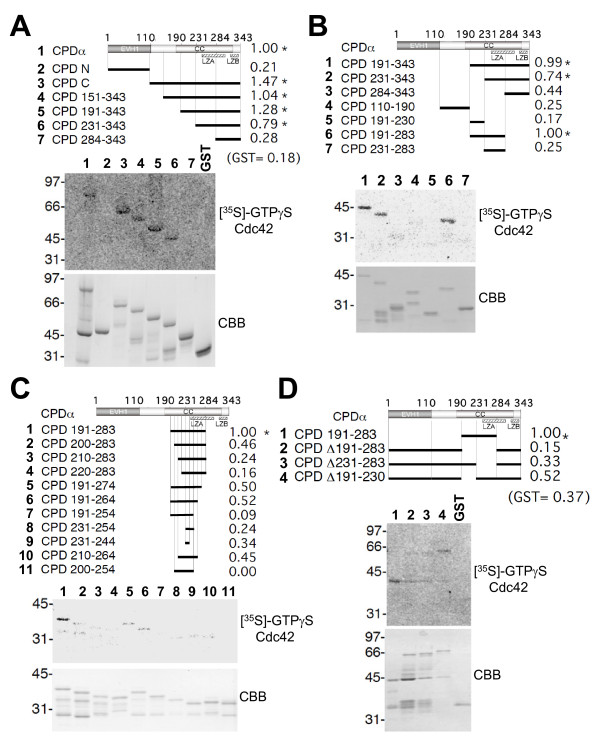
**Determination of the activated Cdc42-binding region of Cupidin and generation of deletion mutants lacking binding activities**. (A-D) Top, the domain structure of Cupidinα/Homer2a (CPDα) and its mutants used in the ligand overlay assay. The Cdc42 binding activities are summarized on the right, presented as normalized values corresponding to CPD (A) or CPD191–283 (B, C, and D) set to 1.00. Asterisks indicate values over 0.70, which was arbitrarily considered to represent positive values. CPDα is composed of 343 amino-acid residues. The EVH1 domain in the N-terminal 110 residues, the coiled-coil domain (CC) predicted within the amino acid stretch 173–317, and two Leu zipper motifs A (LZA, 238–296 aa) and B (LZB, 311–339 aa) are indicated. The deletion constructs consist of the regions shown by solid thick bars; Middle, autoradiograms resulting from the [^35^S]-GTPγS-bound Cdc42 ligand overlay assay; Bottom, Coomassie Brilliant Blue (CBB)-stained gel corresponding to the ligand overlay assay, in which all GST-fused CPD-relating proteins migrated as top bands by SDS-PAGE. (A) First trial to determine the Cdc42-binding region using rough deletion of CPD C. (B) Second trial to narrow down the Cdc42-binding region by making mutants of CPD 191–283. (C) Third trial to narrow down the Cdc42-binding region by using shortened fragments of CPD 191–283. (D) Final trial to determine the deletion mutants of Cupidin lacking Cdc42-binding ability.

Cupidin/Homer2 is comprised of an N-terminal EVH1 domain, a C-terminal coiled-coil (CC) motif and two Leu zipper motifs A and B (LZA and LZB, respectively) (Fig. 1). Our previous study indicated that Cupidin interacts with Cdc42 small GTPase in a GTP-dependent manner via the C-terminal region [6]. To narrow down the region responsible for Cdc42 binding activity, we generated a series of glutathione S-transferase (GST) fusion constructs containing various regions of Cupidin (CPD), as shown in Fig. 1. These GST fusion proteins were blotted onto membrane filters and probed by a ligand overlay assay with GST-fused Cdc42 in the presence of [^35^S]-GTPγS. As described previously [6], GST-CPD and GST-CPD C (C-terminal amino acids (aa) 111–343), but not GST-CPD N (N-terminal aa 1–110), were radioactively labeled (Fig. 1A). No specific radioactive labeling of GST-CPD C was observed in the presence of [^35^S]-GDPβS and Cdc42, as previously reported [6].

Serial deletions starting every 40 aa from amino acid position 111 of CPD C (CPDΔ111–343, 151–343, 191–343, 231–343 and 284–343) showed that the first four deletion mutants, but not the shortest mutant CPD 284–343, retained Cdc42 binding activity (Fig. 1A). Because CPD 191–343 showed high radioactivity and CPD 231–343 showed low radioactivity, we made three further deletion mutants in the region aa 191–343 (CPD 191–230, CPD 191–283 and CPD 231–283) (Fig. 1B). As a result, CPD 191–283 showed high radiolabeling, but neither CPD 191–230 nor CPD 231–283 showed significant labeling, suggesting that the region between amino acids 191 and 283 is necessary for Cdc42 binding. We further N-terminally and C-terminally deleted the region 191–283 to generate 10 deletion mutants and obtained radiolabeling from only the parental CPD 191–283, but not from any of the mutants, indicating that the Cdc42-binding domain resides in the region 191–283 (Fig. 1C). The region 191–283 contains a part of the upstream Leu zipper motif LZA. Thus, we generated three internal deletion constructs: CPDΔ191–283 (deletion of aa 191–283) and CPDΔ231–283 (deletion of aa 231–283) lacked LZA but contained the downstream Leu zipper motif LZB, while CPDΔ191–230 (deletion of aa 191–230) harbored both LZA and LZB (Fig. 1D). CPDΔ191–230 showed radiolabeling that tended to be lower than that of CPD 191–283, whereas CPDΔ191–283 and CPDΔ231–283 showed no significant radiolabeling.

**Figure 2:**
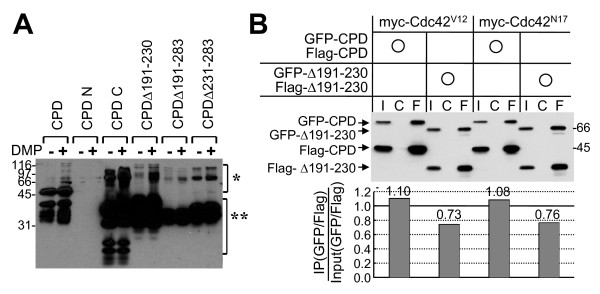
**Multimerization assays with the Cupidin deletion mutants**. Cross-linking assay using the chemical cross-linker DMP. Bacterially expressed GST-proteins were digested by thrombin, and the resulting untagged proteins were incubated in the presence (+) or absence (-) of DMP followed by Western analysis using anti-Cupidin (CPD C) antibody. One asterisk indicates the migration of multimers, and two asterisks indicate that of monomers. (B) Co-immunoprecipitation assay using extracts from cells in which the three distinct constructs shown in the upper panels were heterologously overexpressed in COS7 cells. I, Input; C, IP samples using non-immune serum; F, IP samples using anti-Flag antibody. Western blot analysis was performed using anti-Cupidin antibody, and the multimerization efficacies were calculated from the band signal intensities, and shown in the graph at the bottom.

To assess the multimerization ability of these Cupidin deletion mutants, the GST-proteins were treated with thrombin to remove the GST moiety, which are known to bind each other, and was subjected to a mobility shift assay using sodium dodecyl sulfate-polyacrylamide gel electrophoresis (SDS-PAGE) after treatment with the cross-linker dimethyl pimalidate (DMP). Upon DMP treatment, immunoreactivity for CPDΔ191–230 containing both LZA and LZB was enhanced at a position indicative of multimers in comparison with that for CPDΔ191–283 and CPDΔ231–283, both of which contained LZB only (Fig. 2A). To test a possible correlation between multimerization and Cdc-42 binding ability, we coexpressed both GFP- and Flag-tagged constructs of CPD or CPDΔ191–230 in COS cells together with either dominant-active Cdc42 (Cdc42^V12^) or dominant-negative Cdc42 (Cdc42^N17^), followed by evaluation of the interaction between GFP- and Flag-tagged constructs using immunoprecipitation with an anti-Flag antibody (Fig. 2B). With both CPD and CPDΔ191–230 constructs, GFP-tagged constructs were coimmunoprecipitated with Flag-tagged constructs in intact cells, regardless of whether they expressed active or inactive Cdc42. Although the coimmunoprecipitation efficiency of GFP-tagged CPDΔ191–230, which showed a loss of Cdc42-binding activity, was 30% lower than that of GFP-tagged CPD, these results indicated that Cdc42 binding to Cupidin is substantially independent of the self-multimerization of Cupidin.

### The Cupidin-Cdc42 interaction influences actin-cytoskeletal organization and the morphology of HeLa cells

**Figure 3:**
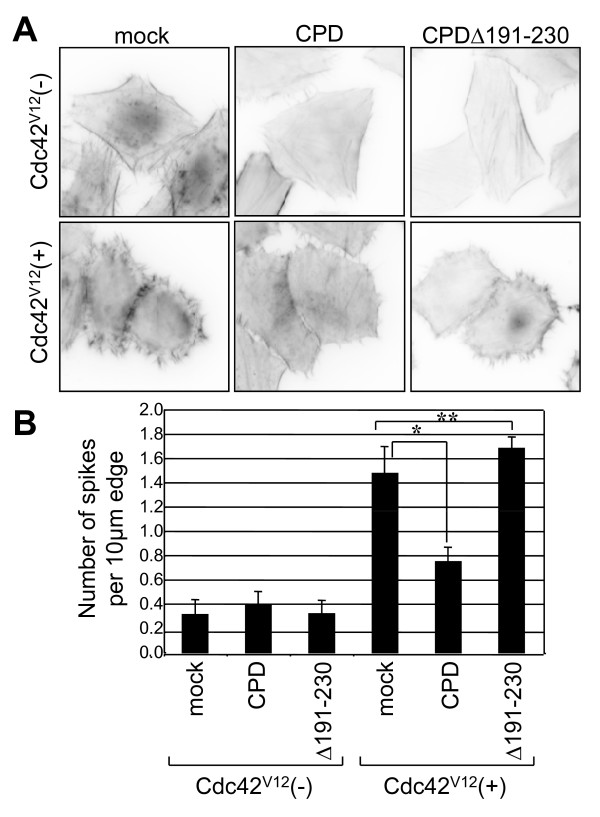
**Cupidin influences Cdc42^V12^-induced changes in HeLa cell morphology**. (A) Human fibroblast HeLa cells were transfected with vector alone (mock), CPD, CPDΔ191–230 (= Δ191–230), Cdc42^V12 ^(dominant active form), Cdc42^V12 ^+ CPD, and Cdc42^V12 ^+ CPDΔ191–230. Actin cytoskeletons of transfected cells were stained by Texas Red-conjugated phalloidin. In mock-, CPD- and CPDΔ191–230-transfected cells, F-actin is concentrated in the cell periphery and in fine stress fibers that traverse in the cytoplasm. On the other hand, cells transfected with dominant-active Cdc42^V12 ^show many filopodia-like protrusions and thin actin bundles around the cell periphery and lack cytoplasmic stress fibers. Co-expression of CPD suppresses Cdc42^V12^-induced filopodia formation, whereas that of CPDΔ191–230 does not. (B) Filopodia formation in (A) was statistically quantified as described in the methods. *, p < 0.01; **, p < 0.001.

We previously showed that co-expressed Cupidin suppresses the dominant-active Cdc42-induced morphological and actin-cytoskeletal changes in HeLa cells [6]. To verify the cellular function of Cdc42 binding, we analyzed the influence of Cdc42-binding deficiency on these cellular phenotypes (Fig. 3A). In HeLa cells transfected with CPD full-length or CPDΔ191–230, stress fibers and bundles of F-actin were formed in the cytoplasm and the cell periphery, respectively, as in cells transfected with vector alone (mock). Upon expression of Cdc42^V12^, a dominant-active form of Cdc42, many filopodia-like protrusions with actin bundles were drastically induced around the cell periphery. Co-expression of the CPD diminished the number of Cdc42^V12^-induced protrusions and actin bundles in the cell periphery. By contrast, co-expression of CPDΔ191–230 did not affect Cdc42^V12^-induced cellular phenotypes, as statistically proven by counting spike number around the cell periphery (Fig. 3B). These results indicated that the region containing amino acids 191–230 is required for the suppression of Cdc42^V12^-induced morphological and actin-cytoskeletal changes in HeLa cells.

### The Cdc42-binding domain of Cupidin is involved in the formation of dendritic spines and synapses in hippocampal neurons

**Figure 4:**
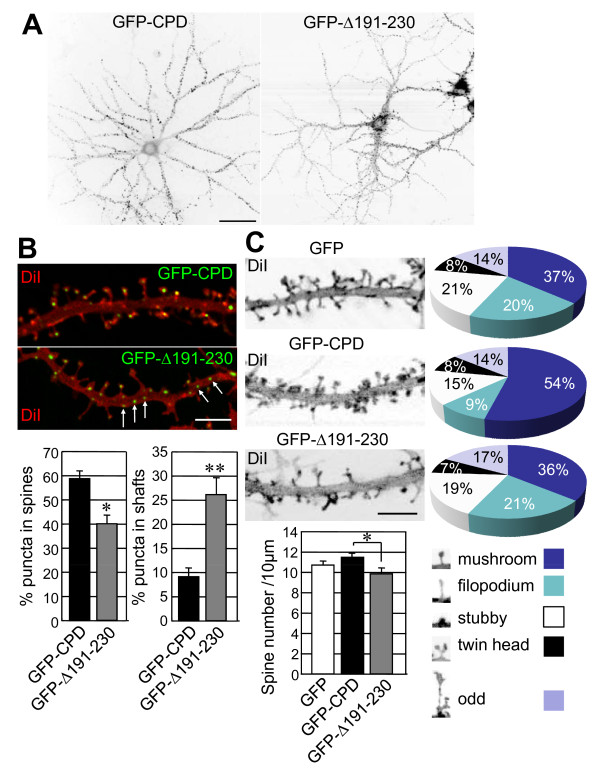
**Deletion of the Cupidin Cdc42-binding domain influences dendritic spine morphology in hippocampal neurons**. (A) GFP fluorescence images of primary cultured rat hippocampal neurons infected with the recombinant adenovirus vector containing GFP-CPD or GFP-CPDΔ191–230 (= GFP-Δ191–230). (B) Dendrites expressing GFP-CPD or GFP-CPDΔ191–230 (= GFP-Δ191–230) fluorescence (green) were outlined by staining with DiI (red). White arrows indicate representative puncta of GFP-CPDΔ191–230 (= GFP-Δ191–230) located in dendritic shafts. The ratios of puncta present in either spine heads or dendritic shafts per total puncta in dendrites were quantified. *, p < 0.01; * *, p < 0.001. (C) Spine morphologies of neurons expressing GFP, GFP-CPD, or GFP-CPDΔ191–230 (= GFP-Δ191–230) were arbitrarily categorized into five types (mushroom-, filopodium-, stubby-, twin head-, and odd-shaped types) as indicated in the inset; each population (%) is shown in the pie charts. The graph shows the number of spines present in a 10-μm length of secondary dendrites of these neurons. *, p < 0.05. Bars: (A) 20 μm, (B and C) 5 μm.

Cupidin is predominantly localized in the dendritic spines of cultured hippocampal neurons [12,23]. To investigate the role of Cdc42 binding in the postsynaptic targeting of Cupidin, we infected primary hippocampal cell cultures with recombinant adenovirus vectors containing enhanced green fluorescent protein (GFP)-fused CPD, GFP-fused CPDΔ191–230 and GFP alone. Punctate fluorescence of GFP-CPD and GFP-CPDΔ191–230 was observed throughout the dendrites of hippocampal neurons (Fig. 4A). In neurons expressing GFP-CPD, approximately 60% of the total punctate fluorescent signals were located at spine heads, whereas less than 10% of these were detected in dendritic shafts (Fig. 4B). On the other hand, in neurons expressing GFP-CPDΔ191–230, about 40% and 30% of the total fluorescent puncta were localized in spine heads and dendritic shafts, respectively (Fig. 4B). These results suggested that the Cupidin C-terminal region possessing Cdc42 binding activity contributes to the targeting of Cupidin to dendritic spines.

Overexpression of Cupidin induced mushroom-type spines in hippocampal neurons as shown in Fig. 4C. On the other hand, overexpression of GFP-CPDΔ191–230 decreased the number of mushroom-type spines and increased filopodia-like or odd-shaped protrusions, although the total number of dendritic protrusions was only slightly reduced in neurons expressing GFP-CPDΔ191–230 compared with neurons expressing GFP-CPD and GFP alone (Fig. 4C). These results are consistent with the idea that Cdc42-binding domain of Cupidin is important for spine morphogenesis and/or maturation.

**Figure 5:**
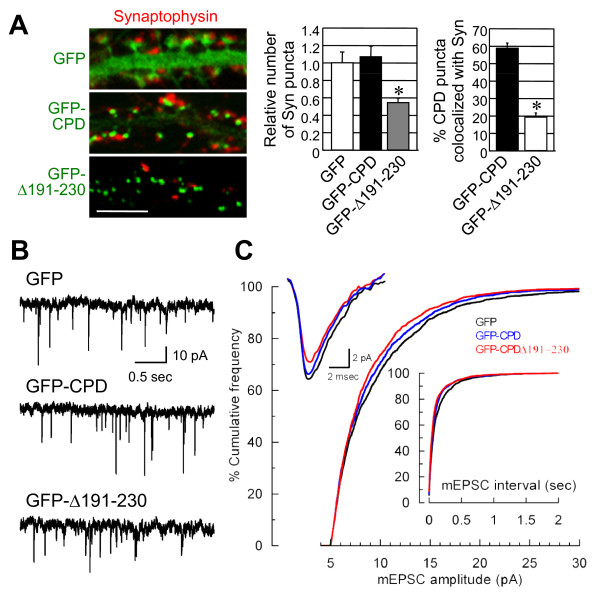
**Deletion of the Cupidin Cdc42-binding domain influences synapse formation and function in hippocampal neurons**. (A) The left panel shows secondary dendrites of hippocampal neurons expressing GFP, GFP-CPD and GFP-CPDΔ191–230 fluorescence (green) following recombinant adenovirus infection were immunostained for synaptophysin (red). The middle graph indicates the relative numbers of synaptophysin-positive puncta along the dendrites overexpressing either GFP-CPD or GFP-CPDΔ191–230 (= GFP-Δ191–230) were quantified and normalized to those of dendrites overexpressing GFP alone, which was set to 1.0 The right graph shows the proportions of GFP-CPD- and GFP-CPDΔ191–230-positive puncta that were co-localized with synaptophysin puncta. *, p < 0.001. Bar: 5 μm. (B) Neurons were infected with the adenovirus vectors containing GFP-CPD, GFP-CPDΔ191–230 (= GFP-Δ191–230) or GFP alone (see Methods) and their ionotropic receptor-mediated mEPSCs were recorded at DIV21. Example recordings of mEPSCs obtained from neurons expressing GFP only (upper trace), GFP-CPD (middle trace), and GFP-CPDΔ191–230 (lower trace). (C) Cumulative frequency plots of mEPSC peak amplitude and interval in neurons expressing GFP only (black line), GFP-CPD (blue line) or GFP-CPDΔ191–230 (red line). Insert: overlapped average of 100 mEPSCs for each construct.

The effect of Cdc42 binding to Cupidin on synapse formation was analyzed by immunostaining for synaptophysin, a presynapse marker (Fig 5A). The number of synaptophysin-positive puncta was drastically reduced in adjacent to dendrites of neurons overexpressing GFP-CPDΔ191–230. In addition, the number of synaptophysin puncta localized adjacent to GFP-CPDΔ191–230 puncta was also decreased. We further explored synaptic functions by measuring the peak amplitudes and frequencies of miniature excitatory postsynaptic currents (mEPSCs) in neurons overexpressing these constructs at 21 DIV (Fig. 5B and 5C). While the amplitudes of mEPSCs in neurons overexpressing GFP-CPD (-9.26 ± 0.12 pA) did not significantly differ from those in neurons overexpressing GFP (-9.86 ± 0.15 pA, p > 0.04), the mEPSC amplitudes in neurons overexpressing GFP-CPDΔ191–230 were significantly reduced (-8.89 ± 0.11 pA, p < 0.001). The mEPSC frequency was enhanced by overexpression of either GFP-CPD (6.90 ± 0.81 Hz, p < 0.001) or GFP-CPDΔ191–230 (6.68 ± 0.94 Hz, p < 0.001) in comparison with overexpression of GFP alone (6.13 ± 0.77 Hz), although at present we could not indicate whether this increased frequency reflects an alteration in the presynaptic release probability or the number of functional synapses. Taken together, these results suggested that a deletion in the Cdc42-binding domain disturbs not only synapse formation, but also synapse electrophysiology.

### The Cupidin-Cdc42 domain is involved in Drebrin targeting into dendritic spines

**Figure 6:**
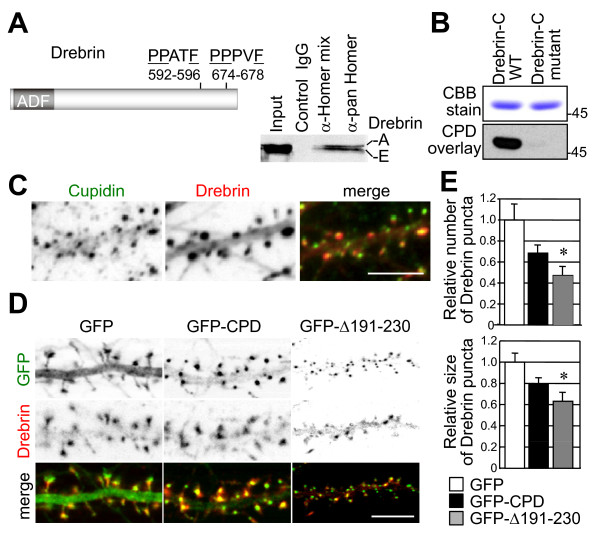
**Cupidin-Drebrin interaction and colocalization in the spines of hippocampal neurons, and its inhibition by expressing a mutant with a deletion of the Cupidin Cdc42-binding domain**. (A) Mouse Drebrin contains an actin depolymerization factor/cofilin-like domain (ADF) in the N-terminal region and two putative PPxxF Homer-binding motifs (592-PPPPATF-596 and 674-PPPVF-678) in the C-terminal region. Both Drebrin-A and Drebrin-E were co-immunoprecipitated with anti-Homer1, 2 and 3 antibodies (Homer-mix) and an anti-pan Homer antibody that recognizes all three Homer family members (right panel). Immunoprecipitates with control rabbit IgG showed no Drebrin bands. (B) The GST-fused mouse Drebrin A C-terminal region (Drebrin-C WT, aa 579-706 containing two Homer ligand motifs 592-PPATF-596 and 674-PPPVF-678) and its double mutant (Drebrin-C mutant with P593A and P675A) were bacterially expressed, electrophoresed and transferred to a nitrocellulose membrane followed by an overlay assay with GST-CPD protein extracts (see Methods). Drebrin-Cupidin binding was proven by Western blot analysis using anti-Cupidin (CPD C) antibody. Top panel, CCB stain; bottom panel, Western blot. (C) Immuno-fluorescent staining of endogenous Cupidin (green) and Drebrin (red) proteins colocalized in the dendritic spines of primary cultured rat hippocampal neurons. Bar: 5 μm. (D) GFP fluorescence (green) and Drebrin-immuno-fluorescence (red) images in the dendrites of hippocampal neurons overexpressing GFP, GFP-CPD or GFP-CPDΔ191–230 (= GFP-Δ191–230) by recombinant adenovirus infection. Bar: 5 μm. (E) The relative puncta number (top graph) and size (bottom graph) of Drebrin-positive puncta along dendrites overexpressing either GFP-CPD or GFP-CPDΔ191–230 (= GFP-Δ191–230) were quantified and normalized to those of dendrites overexpressing GFP alone, which was set to 1.0. *, p < 0.01.

A homology search with the Homer ligand PPxxF motif identified two homologous sites (aa 592–596 and 674–678) in the C-terminal region of mouse Drebrin (Fig. 6A). Coimmunoprecipitation with anti-Homer antibodies (either anti-Homer 1, 2 and 3 antibody mixture or anti-pan-Homer antibody) revealed an association between Homer proteins and Drebrin proteins (two splicing variants A and E) in P8 mouse brains (Fig. 6A). Since the anti-Drebrin monoclonal antibody used recognizes the C-terminal region near the PPxxF motifs, the anti-Drebrin antibody might interfere with the Cupidin-Drebrin interaction in a reverse coimmunoprecipitation of Cupidin with anti-Drebrin antibody. Thus, we carried out an overlay assay in which Cupidin was tested for binding to a polypeptide containing two Drebrin PPxxF motifs (aa 579–706 of mouse Drebrin A) or its double mutant (P593A and P675A) blotted onto a membrane filter (Fig. 6B). Cupidin bound to the PPxxF-containing polypeptide, but not to the mutant polypeptide, again indicating that Cupidin interacts with the C-terminal PPxxF motifs of Drebrin.

Endogenous Cupidin and Drebrin were both punctately distributed in dendritic spines of immunostained primary hippocampal neurons (Fig. 6C). Cupidin puncta (which are known to concentrate in the PSD [6,12]) largely overlapped with Drebrin puncta (which are known to concentrate throughout spine heads [21]) around the bottom half of spine heads. We next analyzed the effects of GFP, GFP-CPD, or GFP- CPDΔ191–230 overexpression on the dendritic distribution of Drebrin in primary hippocampal neurons (Fig. 6D and 6E). The number of Drebrin puncta was slightly reduced by overexpressing Cupidin, but was significantly reduced by overexpressing GFP-CPDΔ191–230 (Fig. 6D and 6E). Taken together, these results suggested that a deletion in the Cdc42-binding region of Cupidin disturbs dendritic Drebrin distribution in hippocampal neurons.

## Discussion

**Figure 7:**
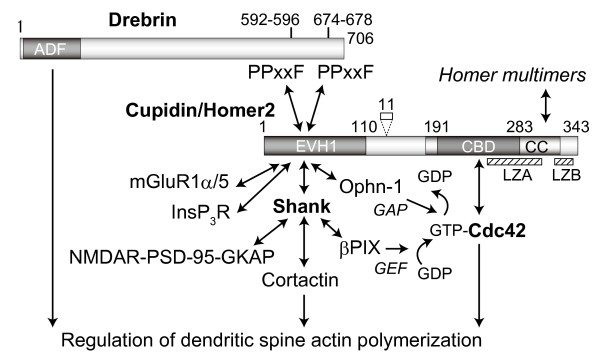
**Cupidin/Homer scaffolds for actin cytoskeleton modulators and postsynaptic proteins**. Cupidin/Homer2 acts as a scaffold tethering postsynaptic signalling proteins (such as mGluR1α/5, IP_3_R, and Shank) as well as the dendritic actin-binding protein Drebrin, a spine actin modulator, via the N-terminal EVH-1 domain, which recognizes the PPxxF consensus motif. It also links the GTP-bound, activated Cdc42 small GTPase, which is known to regulate filopodia formation, via the C-terminal CBD (Cdc42-binding domain) and forms a tetrameric scaffold complex via the C-terminal coiled-coil (CC) and Leu zipper motif (LZA and LZB) regions. The Homer scaffold complex is implicated in the facilitation of signalling crosstalk among tethered synapse proteins and regulation of the actin-based morphology of dendritic spines.

Our study demonstrates the structural and functional interaction of Cupidin/Homer2 with two dendritic spine F-actin modulators, Cdc42 small GTPase, via the C-terminal region, and Drebrin, via the N-terminal EVH1 domain. Cdc42 regulates actin polymerization and is involved in filopodia formation [24] and dendritic spine morphogenesis [18-20]. Over-expression of Drebrin increases spine length [21] and promotes synaptic clustering of PSD-95 and F-actin [22]. Homer also interacts with Shank, another postsynaptic scaffold protein that binds to the GKAP/PSD-95/NMDA receptor complex [9]. Overexpression of Shank together with Homer induces enlargement of spine heads [13] and increases the level of the βPIX guanine nucleotide exchange factor (GEF) for Cdc42 in the PSD [13,25]. Moreover, oligophrenin-1 (Ophn-1), a Rho GTPase activating protein (GAP) that is involved in non-specific X-linked mental retardation and binds Homer1b/c, changes spine morphology in hippocampal neurons [26]. Taken together, these lines of evidence suggest that postsynaptic Homer-mediated scaffolding is involved in the regulation of dendritic spine morphology by interacting with the actin organization signaling molecules Cdc42 and Drebrin, as well as synaptic signaling molecules including the NMDA receptor complex, mGluR1α/5 subunits and InsP_3_R (Fig. 7).

We defined the Cdc42-binding domain (CBD) as the 96 amino acid residues in positions 191–283 within the C-terminal CC region. Among Homer family members, there are fragmentary sequence similarities (15 identical amino acids and 15 functionally similar amino acids) in the CBD region (Additional file 1). Homer interacts with Ophn-1 GAP [26], whereas Shank interacts with βPIX GEF [25], as described above. Thus, Homer-Shank scaffolds linking these GAP and GEF activities may synergistically regulate the activation (GTP-bound state) and inactivation (GDP-bound state) of Cdc42, which is involved in actin cytoskeleton regulation via the N-WASP, IRSp53-WAVE or PAK signaling pathways [24], resulting in fine regulation of spine morphology.

The N-terminal EVH1 domain of Homer recognizes the Homer ligand motif PPxxF [27-30]. Homer-binding target proteins identified thus far have only one PPxxF motif, except that TRPC1 (a transient receptor potential cation channel member) has two Homer binding sites, referred to as type 1 (PPxxF or PxxF) and atypical type 2 (LPSSP) [31]. Intriguingly, mouse, rat and human Drebrin proteins (splice variants Drebrin A and Drebrin E) possess two conserved type 1 Homer ligand motifs (ligand-1: PPATF and ligand-2: PPPVF) in the C-terminal region (Additional file 2). However, the truncated variant s-Drebrin has no motif. These lines of evidence suggest that the Cupidin/Homer2-Drebrin interaction is regulated by expression of these various forms. Chick and *Xenopus *Drebrin have only Homer ligand-1, although their motif sequence (PPATF) is identical to that of mouse, rat and human. An association of decreased levels of Drebrin with deterioration of spines and synapses was reported in the hippocampal synapses [32,33] and brains [32,33] of patients with Alzheimer's disease (AD) as well as in Aβ peptide-treated hippocampal neurons [34]. Thus, the interaction between Cupidin/Homer2 and Drebrin may be associated with the changes in spine morphology found in individuals with AD.

## Conclusion

Cupidin/Homer2 interacts with activated Cdc42 via the Cdc42-binding domain within the C-terminal coiled-coil domain, which may play a role in the suppression of Cdc42-induced filopodia-like protrusion formation in HeLa cells and the formation of mushroom-type spines in hippocampal neurons. Cupidin/Homer2 interacts with a dendritic spine F-actin-binding protein Drebrin via the N-terminal EVH-1 domain. Drebrin possesses two Homer ligand motifs in the C-terminal region, and is mostly colocalized with Cupidin around the spine heads. Drebrin clustering in dendritic spines is disturbed by overexpression of Cupidin deficient in Cdc42 binding. These results suggest that Cupidin/Homer2 is involved in the modulation of spine morphology and function by scaffolding multiple target proteins, including the two dendritic spine actin regulators Cdc42 small GTPase and Drebrin.

## Methods

### Construction and expression of GST fusion proteins in *E. coli*

Glutathione S-transferase (GST) fusion constructs were generated by cloning various parts of Cupidinα/Homer2a cDNA [6] into the GST fusion vector pGEX-KG (see, Fig. 1). *Escherichia coli *JM109 expressing GST-fusion proteins were lysed in lysis buffer (50 mM Tris-HCl pH 7.4, 25% sucrose, 1% Triton X-100, 5 mM MgCl_2_). GST fusion protein lysates (10 mg) were coupled to glutathione-Sepharose 4B (Amersham Pharmacia Biotech, Piscataway, NJ) by rotating for 1 h at 4°C. After washing three times with 1% Triton X-100/phosphate-buffered saline, GST fusion protein-coupled Sepharose was mixed with 1 mg of protein lysates prepared from mouse cerebella, which were pre-cleared with glutathione-Sepharose for 1 h at 4°C. After rotating for 1 h at 4°C, the GST fusion protein complex was washed five times with cell lysis buffer and subjected to immunoblotting.

### Ligand overlay assay with Cdc42

Bacterially expressed GST-Cdc42 protein was purified using glutathione-Sepharose column chromatography according to a previously described procedure [6]. One μg samples of non-degraded GST-fusion proteins were separated by 10% SDS-PAGE and blotted onto nitrocellulose membranes (Hybond-ECL; Amersham Pharmacia Biotech, Piscataway, NJ). A ligand overlay assay was carried out as described previously [6]. Briefly, after the GST-fusion proteins on the blots were re-natured, the protein blots were probed by incubating with each GST-Rho family fusion protein loaded with [^35^S]-GTPγS at an equal specific activity. After washing three times, the ligand-bound blots were air-dried and the radioactivities were analyzed using a BAS2000 Bioimaging analyzer (Fujix, Japan). The relative radioactivities were respectively measured from consistently sized areas using IPLab software (Scanalytics, Fairfax, VA), and normalized as described in the figure legends (Fig. 1).

### Western blot analysis

After boiling proteins in sample buffer (0.4 M Tris-HCl pH 6.8, 8% sodium dodecyl sulfate, 40% (v/v) glycerol, 0.04% bromophenol blue) for 5 min, equal portions of protein solution were separated by SDS-PAGE and electro-transferred onto nitrocellulose membrane filters (GE Healthcare). Blots were reacted with diluted primary antibodies: anti-Cupidin antibody (1:5000) [12], anti-pan Homer antibody (1:1000) [12], anti-Drebrin antibody (1:400) (D029-3, MBL), anti-Flag monoclonal antibody (1:1000) (F3165, Sigma), anti-GFP antibody (1:400) (11814460001, Roche), anti-Myc monoclonal antibody (1:1000) (sc-40, Santa Cruz). Immunoreactivity was detected with ECL (GE Healthcare).

### Cross-linking assay

Bacterially expressed GST-CPD, GST-CPD N, GST-CPD C, GST-CPDΔ191–230, GST-CPDΔ191–283, and GST-CPDΔ231–283 proteins were digested with thrombin to remove the GST moiety, and dialyzed against a crosslinking buffer (10 mM HEPES-NaOH, pH 7.5, 2 mM EDTA, 1 mM MgCl_2_, 0.05% Tween 20, 5 mM DTT, and 1 mM GDP). Each GST- protein (25 μg/ml) was incubated with 10 mM dimethyl pimelimidate (DMP) (Pierce, Rockford, IL) for 1 hr at room temperature. Equal amounts of DMP-treated protein mixtures were analyzed by Western blotting using anti-CPD antibody.

### Co-immunoprecipitation

For examination of the effects of Cdc42 binding on Cupidin multimerization, COS7 cells were triple-transfected with Flag-tagged CPD, GFP-tagged CPD and either myc-tagged Cdc42^V12 ^or myc-tagged Cdc42^N17^. Similarly, COS7 cells were triple-transfected with Flag-tagged CPDΔ191–230, GFP-tagged CPDΔ191–230, and either myc-tagged Cdc42^V12 ^or myc-tagged Cdc42^N17^. To prepare protein extracts from these transfected cells, cells were lysed and homogenized in 1% Triton X-100 buffer (50 mM HEPES, 150 mM NaCl, 10% glycerol, 1% Triton X-100, 1.5 mM MgCl_2_, 1 mM EGTA, 100 mM NaF, 1 mM Na_3_VO_4_, 10 μg/ml aprotinin, 10 μg/ml leupeptin, and 1 mM phenylmethylsulfonyl fluoride). After centrifuging at 14,000 × g for 10 min, protein solutions (containing approximately 1 mg proteins) were mixed with anti-Flag antibody, and incubated for 1 h on ice. Protein-antibody complex was precipitated with protein G-Sepharose (GE Healthcare) followed by repeated centrifugation at 2000 × g for 5 min at 4°C. The precipitated proteins were subjected to Western blot analysis using anti-CPD antibody. Signal intensities in areas of consistent size were measured using IPLab software, and the efficiency of multimerization was calculated as described in Fig. 2.

For examination of the Cupidin-Drebrin interaction, mouse cerebella (ICR, Nihon SLC, Hamamatsu, Japan) were lysed and homogenized in 1% Triton X-100 buffer (50 mM HEPES, 150 mM NaCl, 10% glycerol, 1% Triton X-100, 1.5 mM MgCl_2_, 1 mM EGTA, 100 mM NaF, 1 mM Na_3_VO_4_, 10 μg/ml aprotinin, 10 μg/ml leupeptin, and 1 mM phenylmethylsulfonyl fluoride). After centrifuging at 14,000 × g for 10 min, protein solutions (containing approximately 1 mg proteins) were mixed with primary antibody (non-immune serum, anti-CPD C antibody, or anti-pan Homer antibody), and incubated for 1 h on ice. Protein-antibody complex was precipitated with protein G-Sepharose (GE Healthcare) followed by repeated centrifugation at 2000 × g for 5 min at 4°C. The precipitated proteins were subjected to Western blot analysis using anti-Drebrin antibody.

### Cell morphology of transfected HeLa cells

HeLa cells were transfected with CPD alone, CPDΔ191–230 alone, Cdc42^V12 ^alone, Cdc42^V12 ^and CPD, or Cdc42^V12 ^and CPDΔ191–230 using the calcium phosphate precipitation method described previously [6]. At 24 hours after transfection, cells were fixed with 4% formalin in PBS and stained with Alexa Fluor568-conjugated phalloidin (1:1000) (A12380, Invitrogen). Fluorescence was observed with a microscope (Eclipse E800; Nikon, Tokyo, Japan) equipped with a CCD camera (SPOT; Diagnostics Instruments Inc., Sterling Heights, MI). The phalloidin images were captured following confirmation of completing single/double transfection by detection of distinct fluoroprobes, as described by Shiraishi et., al [6]. The number of spikes protruding from the cellular edge were counted in 10 cells respectively, and represented as the means ± SE per 10 μm of cell edge; data were compared by a two-tailed unpaired Student *t *tests using Excel software (Microsoft Corporation, Tokyo, Japan).

### Preparation of primary hippocampal cell cultures

Hippocampal primary cell cultures were prepared from embryonic day 17 Wistar rats (Nippon SLC, Shizuoka, Japan) as described previously [12]. Briefly, hippocampi were dissected after rats had been anesthetized with diethyl ether; excised hippocampi were treated with 45 U of papain (Worthington, PAPL, Lakewood, NJ), 0.01% DNase I (Boehringer-Mannheim, Indianapolis, IN), 0.02% DL-cysteine, 0.02% bovine serum albumin, and 0.5% glucose in PBS for 20 min at 37°C. After adding 20% bovine serum, cells were dissociated by repeatedly passing them through a 1-mL plastic pipette tip. Dispersed cells were plated at a density of 1.1 × 10^4 ^cells/cm^2 ^onto poly-L-lysine-coated glass coverslips (Matsunami, Tokyo, Japan) in neurobasal medium (GIBCO BRL, Life Technologies, Rockville, MD) containing 2% B27 supplement (Invitrogen), 500 mM L-glutamine, 0.1 mg/mL streptomycin (Meiji, Tokyo, Japan), and 100 U/mL penicillin (Banyu, Tokyo, Japan). Cultures were maintained in a humidified atmosphere of 5% CO_2 _in air at 37°C.

### Construction of and infection with recombinant adenovirus vectors

The EGFP-coding region (referred as to GFP in this study) derived from pEGFP-C1 (Clontech, Cambridge, UK) was fused in frame to the N-terminus of the full-length or mutated constructs of Cupidin to generate GFP-CPD. The GFP fragment was also fused to Cupidin with a deletion of amino acid residues 191–230 (CPDΔ191–230) to generate GFP-CPDΔ191–230. Replication-deficient adenovirus vectors carrying these GFP-fused constructs were generated by the COS-TPC method, as described previously [35]. Briefly, the DNA fragment of GFP-CPD or GFP-CPDΔ191–230 was inserted into the *Swa*I site of the pAxCAwt cosmid cassette (Takara, Tokyo, Japan). The resultant cosmid DNA was co-transfected with the complex of the *Eco*T22I-digested Ad5-dlx DNA and the terminal protein into HEK293 cells, and recombinant adenoviruses were thus obtained by homologous recombination between them. The viruses were propagated in HEK293 cells, and were concentrated and purified by double CsCl step gradient centrifugation. The titers of viruses were measured by the 50% tissue culture infectious dose (TCID50) method. Hippocampal cultures at 19 days *in vitro *(DIV) were infected with the viruses at a multiplicity of infection (m.o.i.) of 100–200, and were analyzed at post-infection 2 days, corresponding to 21 DIV.

### Analysis of spine morphology

Hippocampal cultures were fixed with 4% formaldehyde for 10 min and directly incubated with Oregon Green phalloidin (Molecular Probes, 1:200) overnight at 4°C. DiI (Molecular Probes) emulsion, mixed with cod liver oil at 1 μg/μl, was put onto the somata of neurons, which were identified by phalloidin staining, as a droplet of 20–30 μm in diameter, using a manually handled injector (Narishige, Tokyo, Japan). After incubation overnight at 4°C, the excess un-penetrated DiI emulsions were removed by suction, and DiI images were captured by confocal microscopy (MRC1024; BioRad, Hercules, CA) with 100×, 1.4 NA lens. Digital images were processed using Adobe Photoshop 6.0 software (Adobe Systems, San Jose, CA). Numbers of either GFP-CPD or GFP-CPD puncta were manually counted on the secondary dendrites of 10 neurons and the results presented as the means ± SE; data were compared by a two-tailed unpaired Student *t *test using Excel software. Spine morphology was categorized into five types as described in the legend for Fig. 4. Over 1,000 protrusions on the secondary dendrites of 20–30 neurons were analyzed for evaluation of spine morphology.

### Immunocytochemistry

All immunocytochemical procedures were performed as described previously [12]. Briefly, primary-cultured neurons (21 DIV) overexpressing GFP-constructs by adenovirus-mediated infection were fixed with 4% paraformaldehyde for 30 min at 37°C, washed three times with PBS, and then permeabilized with 0.2% Triton X-100 in PBS for 10 min. After preincubation with 5% BSA in PBS for 1 h, cells were incubated with primary antibody (anti-synaptophysin or anti-Drebrin antibody) for 1 h at 37°C. After washing three times with PBS, the cells were incubated with Alexa Fluor 568-conjugated anti-mouse IgG (Invitrogen). Fluorescence and phase-contrast images of immunostained cells were captured by confocal microscopy (MRC1024; BioRad, Hercules, CA) to acquire a single focal plane with a 100×, 1.4 NA lens. Digital images were processed using Adobe Photoshop 6.0 software (Adobe Systems, San Jose, CA). The number of punctate immunopositive signals larger than 1 pixel (0.16 × 0.16 μm^2^/pixel with a 255-gradient signal intensity; signals lower than 165 on the scale were cut off to eliminate noise) was counted by measuring the area with a signal above 165 on the scale, using IPLab software. Scores from the secondary dendrites of 10 neurons were normalized to each control (= 1.0). Results presented as mean ± SE were compared by two-tailed unpaired Student *t *tests using Excel software.

### Electrophysiology

Glass coverslips with infected cells (as indicated by GFP fluorescence) at 21 DIV were transferred to an experimental chamber and superfused with modified Krebs-Ringer solution (in mM): NaCl 150, KCl 4, CaCl_2 _2, glucose 5, pyruvate 2, HEPES 5 (pH 7.4 with NaOH). Tetrodotoxin (1 μM) and picrotoxin (50 μM) were added to block action potentials and inhibitory synaptic transmission, respectively. The experimental chamber, consisting of an acrylic frame with a glass bottom, was mounted on the stage of an inverted microscope equipped with interference-contrast optics (Axiovert 100S, ZEISS, Germany). Patch pipettes were pulled from glass capillaries (Clark Electromedical Instruments, Pangbourne, U.K.) with a horizontal puller (P-97 Flaming/Brown Micropipette Puller, Sutter Instrument Company, U.S.A.). The pipettes had direct current resistance of 3–6 MΩ (tip diameter ~1–2 μm) when filled with solution (in mM): K-gluconate 25, KOH 80, CsCl 60, methane sulfonic acid 60, MgCl_2 _4, CaCl_2 _0.8, EGTA 2, Na_2_-ATP 4, Na_2_-GTP 0.2, glutathione 1, glucose 5 and HEPES 30 (pH 7.2 with CsOH, ≅ 330 mosm/l). The pipettes were connected to a patch-clamp amplifier and filtered with a 1-kHz Bessell low-pass filter (AXOPATCH 200B, Axon Instruments, U.S.A.). Data acquisition was done with Clampex software (Axon Instruments, U.S.A.). Miniature EPSCs sampled at 50 kHz were detected and fitted to a template function using custom software [36] written in IDL (Research System Inc., Boulder, CO). Peak amplitudes and interval were calculated for about 200 mEPSCs from each cell. Detection threshold was set to 5 pA amplitude. The data from 12 cells for each construct were compared using the Kolmogorov-Smirnov nonparametric test. Significance was set at p < 0.01. Recordings were performed at room temperature (22–25°C).

## Authors' contributions

YS-Y conceived and carried out most of the molecular and cellular experiments and participated in writing the manuscript. YS participated in some of the experiments using primary hippocampal cell cultures and generated the recombinant adenovirus vectors. RS carried out electrophysiological experiments and analyses. AM conceived and participated in the Drebrin experiments. TK, NM and KM provided useful discussion and contributed to supervision. TF participated in the design and coordination, supervised the study, and wrote the manuscript. All authors read and approved the final manuscript.

## Supplementary Material

Additional file 1 **An amino-acid sequence alignment of the Cdc42-binding domain (CBD) (191–283 residues of Cupidin-α or Homer2a) among three Homer family members, Cupidinα/β (= Homer2a/b), Homer1b/c and Homer3, in mice, is shown.** The 15 amino acids identical among the family members are highlighted by black boxes. Functionally similar amino acids among three or two family members are shown in bold. The Leu zipper A (LZA) and B (LZB) motifs are underlined with a zigzag line and conserved Leu residues are indicated by closed circles. Click here for file

Additional file 2 **Mouse, rat, human and chicken Drebrin have two Homer ligand motifs (PPxxF), Homer ligand 1 and ligand 2, in the C-terminal region, whereas *Xenopus *Drebrin has only one motif.** Click here for file
