# Supplementary figures and images for: Interaction of Cupidin/Homer2 with two actin cytoskeletal regulators, Cdc42 small GTPase and Drebrin, in dendritic spines
Source: BMC Neurosci. 2009 Mar 24;10:25. doi: 10.1186/1471-2202-10-25 (PMC2666743; doi:10.1186/1471-2202-10-25)

Cupidina $\alpha/\beta$  = Homer2a/b:  
Homer1b/c:  
Homer3:

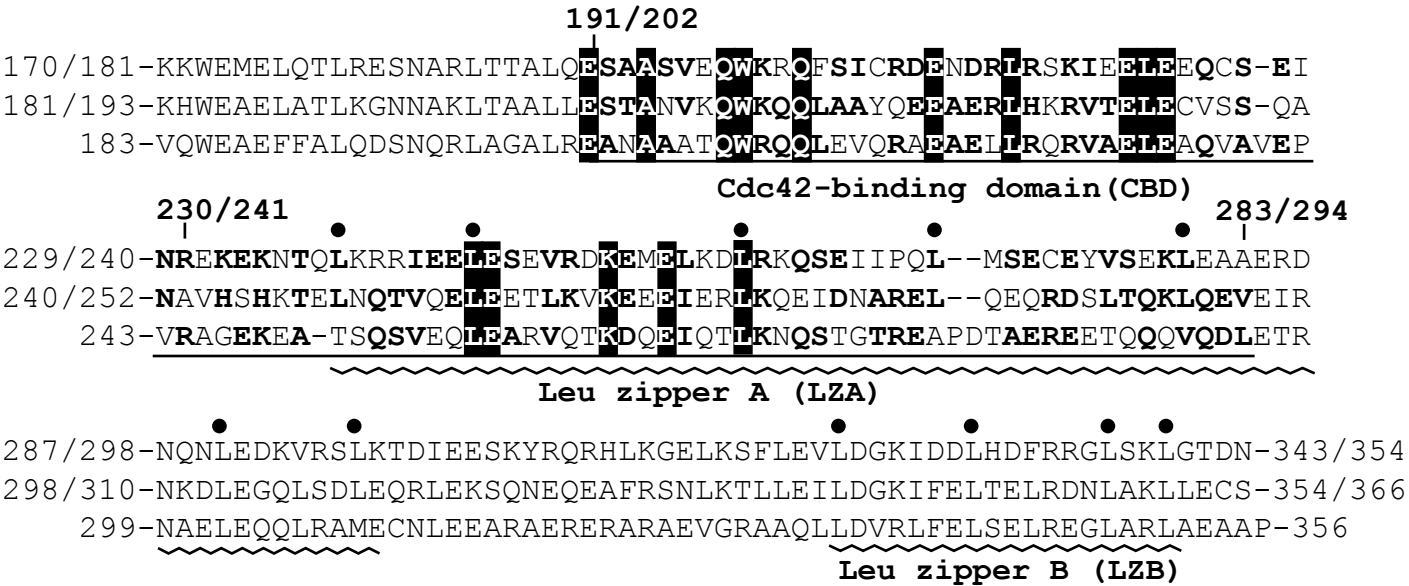

Supplement: Additional file 1 — An amino-acid sequence alignment of the Cdc42-binding domain (CBD) (191–283 residues of Cupidin-α or Homer2a) among three Homer family members, Cupidinα/β (= Homer2a/b), Homer1b/c and Homer3, in mice, is shown. The 15 amino acids identical among the family members are highlighted by black boxes. Functionally similar amino acids among three or two family members are shown in bold. The Leu zipper A (LZA) and B (LZB) motifs are underlined with a zigzag line and conserved Leu residues are indicated by closed circles. [file 1471-2202-10-25-S1.pdf]
