# Supplementary material for: Interaction of Cupidin/Homer2 with two actin cytoskeletal regulators, Cdc42 small GTPase and Drebrin, in dendritic spines
Source: BMC Neurosci. 2009 Mar 24;10:25. doi: 10.1186/1471-2202-10-25 (PMC2666743; doi:10.1186/1471-2202-10-25)
Supplement: Additional file 2 — Mouse, rat, human and chicken Drebrin have two Homer ligand motifs (PPxxF), Homer ligand 1 and ligand 2, in the C-terminal region, whereas Xenopus Drebrin has only one motif. [file 1471-2202-10-25-S2.pdf]

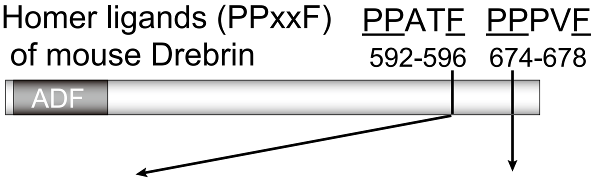

	Homer ligand 1	Homer ligand 2
Mouse	592-**PP**AT**F**-596	674-**PP**PV**F**-678
Rat	**PP**AT**F**	**PP**PV**F**
Human	**PP**AT**F**	**PP**PV**F**
Chicken	**PP**AT**F**	**P**QPV**F**
Xenopus	**PP**AT**F**	**P**LPV**F**
